# A fatal aorto-oesophageal fistula due to a mutton bone: A case report

**DOI:** 10.1016/j.ijscr.2023.108478

**Published:** 2023-07-07

**Authors:** S. Pranavan, U. Mayorathan, B.M. Munasinghe

**Affiliations:** a Teaching Hospital, Jaffna, Sri Lanka; b Department of Anaesthesiology and Intensive Care, Queen Elizabeth the Queen Mother Hospital, Margate, Kent, UK

**Keywords:** Aorto-oesophageal fistula, AEF, Mutton bone, Fatal, Haemorrhage, Food bolus impaction

## Abstract

**Introduction and importance of the case:**

Aorto-oesophageal fistula (AEF) following foreign body ingestion is rare and conservative management is always fatal. The delayed presentation further confounds poor outcomes.

**Presentation of case:**

A 46-year-old South-Asian woman presented with pain and difficulty in swallowing following ingestion of a mutton-containing meal. The patient refused urgent upper GI endoscopy and was initially managed conservatively on the basis of the resolution of symptoms and hemodynamic stability and was discharged home. On review a week later, the patient did not consent to a UGIE. She presented the next day with a severe upper GI bleed. Due to profuse haemorrhage, a bleeding point could not be identified, and she suffered a cardiac arrest. Attempts at resuscitation were unsuccessful. The autopsy revealed an AEF caused by a sharp mutton bone lodged in the lower oesophagus.

**Clinical discussion:**

High-risk food bolus impactions such as the ones caused by sharp objects need urgent endoscopy to confirm the position and extraction if safe. AEF occurs with time and could result in massive haemorrhage and mediastinitis. Endoscopic stenting, thoracoscopic surgery, and open repair are methods of emergent and definite management that still carry significant mortality.

**Conclusion:**

Management of AEF requires early diagnosis with a high index of suspicion, endoscopic and CT-based angiography studies, and surgical interventions tailored to patients based on the available expertise. High-risk patients should be similarly educated on the probable complications and the symptomatology.

## Highlights

• Aorto-oesophageal fistula (AEF) following foreign body ingestion is rare.
• A classical triad of symptoms (retrosternal pain, sentinel bleeding, and massive haemorrhage) has been described.
• Conservative management is universally fatal.
• CT angiography studies could be useful in the diagnosis.
• Immediate and long-term mortality is still high even after operative interventions.

## 1 Introduction

Aorto-oesophageal fistula (AEF) is an anomalous communication between the oesophagus and the aorta. Foreign bodies are rare culprits of AEF [1]. Patients with AEF present with a variety of symptoms and a classical triad of symptoms including retrosternal pain, sentinel bleeding, and life-threatening massive haemorrhage has been described [2]. The outcome is still poor. Conservative management is universally fatal and interventions are associated with an increased mortality of 40–60 % [3], and some authors even state rates up to 80 % [4]. Overall outcomes in gastrointestinal foriegn bodies may be even poorer in low-resource settings [5]. This case presents a fatal outcome following the ingestion of a mutton bone in a middle-aged South Asian woman who refused initial endoscopy and later presented with massive haematemesis. The case report follows SCARE guidance [6].

## 2 Case description

**Fig. 1:**
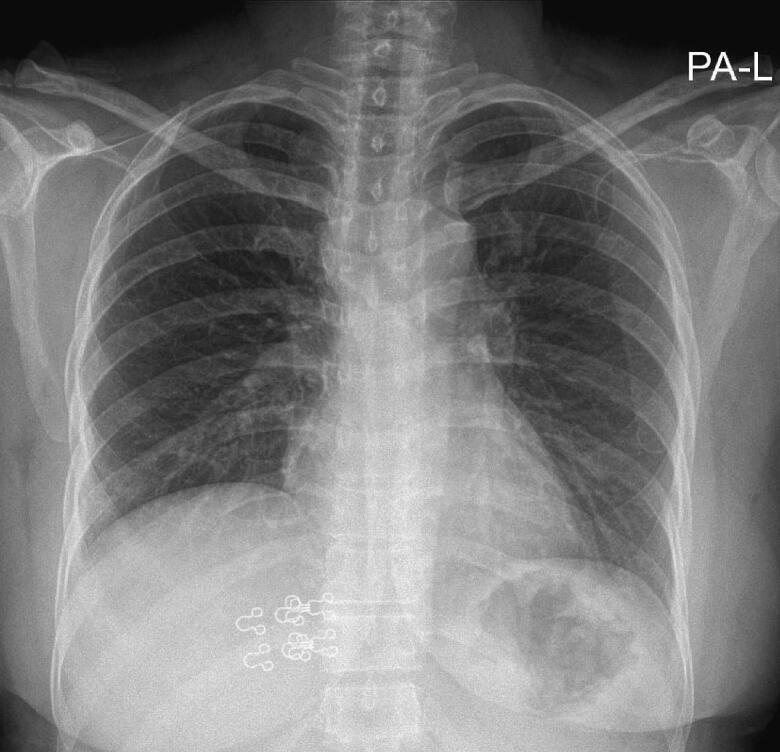
Initial normal chest X-ray.

A 46-year-old South-Asian woman presented to a Tertiary care center in the Northern province of Sri Lanka with difficulty and pain during swallowing, nausea and vomiting, and central chest pain for one day following ingestion of a mutton-containing meal. The pain was progressively worsening. There was no stridor and no focal neurological deficits were noted. She was afebrile, not pale and the vital parameters were stable. She was a diagnosed patient with type 2 diabetes mellitus with good control. The past surgical history and social history were unremarkable. The patient was admitted for further investigations. Basic biochemistry including random blood sugar, ECG, and Troponin T titer were normal. Chest X-ray (Fig. 1) did not show any foreign bodies.

**Fig. 2:**
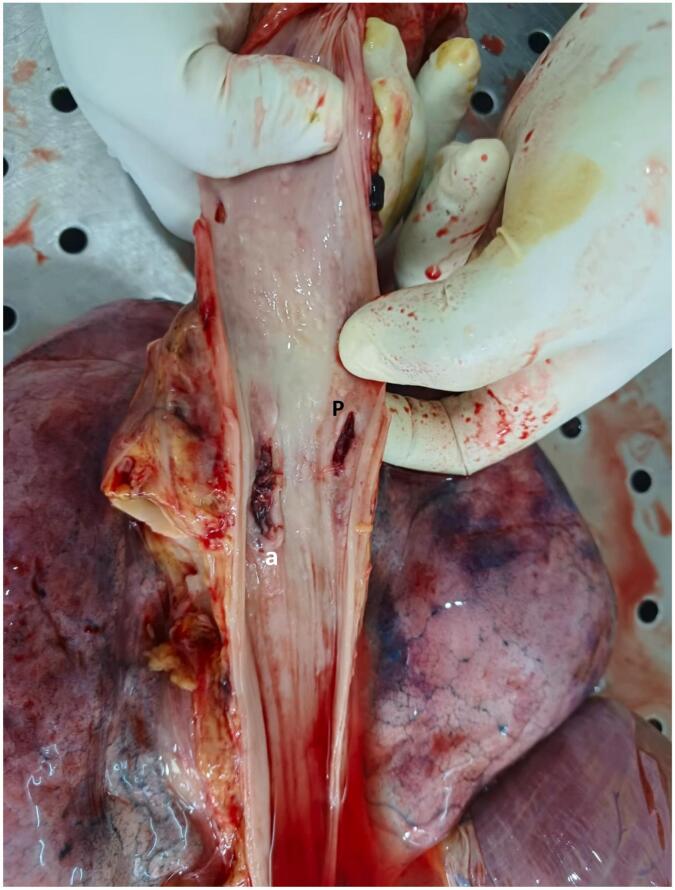
Oesophagus at autopsy. Mutton bone lodged in anterior wall (a); slit-like perforation in posterior wall (P).

**Fig. 3:**
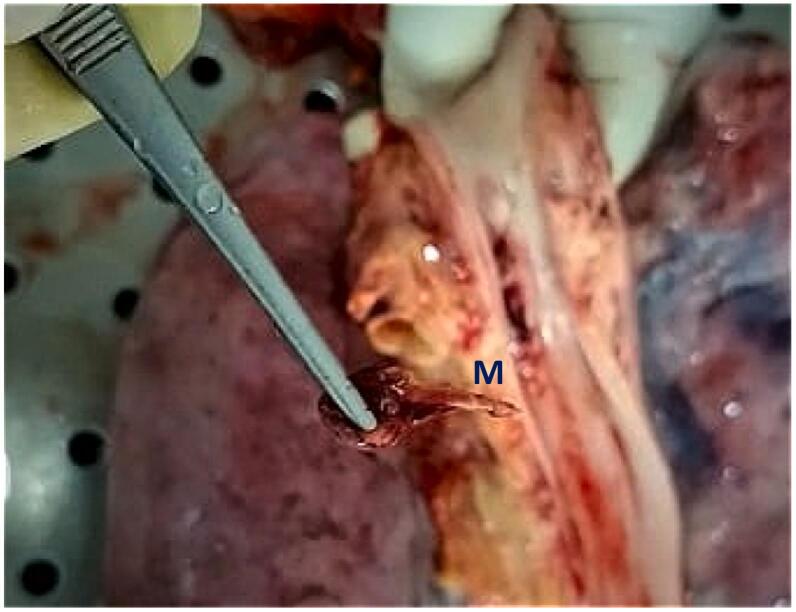
Impacted mutton bone (M) after removal.

**Fig. 4:**
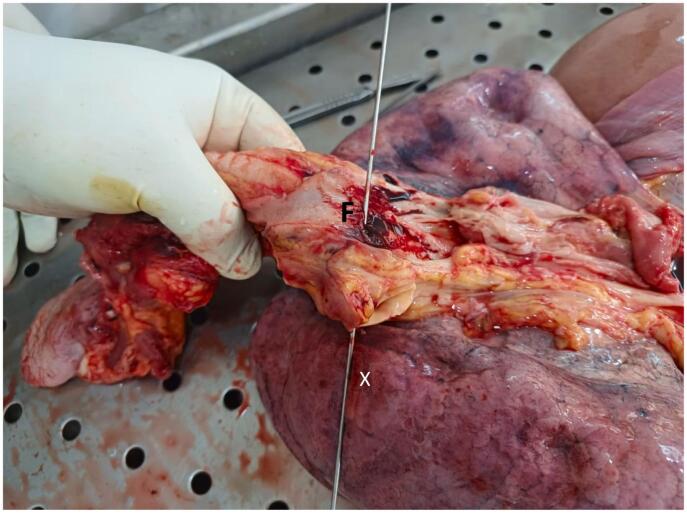
Probe (X) illustrating the AEF (F) below the level of aortic arch.

An urgent upper gastrointestinal endoscopy (UGIE) was planned. However, the patient refused the procedure despite a detailed explanation of possible poor outcomes. She was managed with an intravenous H2 receptor blocker and antiemetics. Oral intake was permitted as the patient admitted to having resolution of symptoms. She was discharged with strict instructions to return for a review in a week or admit urgently prior in case of worsening symptoms. She was reviewed after a week and she denied any symptoms and she did not consent to a UGIE. She developed an episode of haematemesis at home the same day and was admitted to the hospital the next day evening with massive haematemesis and malena. On admission, she was pale and drowsy. The blood pressure was 106/95 mmHg and the pulse rate was 120/min. The haemoglobin level was 85 g/L and the C-reactive protein level was 22 mg/L. Immediate resuscitation was commenced with the activation of major haemorrhage protocol and an urgent UGIE was performed by the consultant gastroenterologist. Due to gross haematemesis, a bleeding point could not be visualized and the patient had an asystolic cardiac arrest. The procedure was abandoned. The patient died shortly after despite ongoing resuscitation before CT or angiography studies. During the autopsy, upon opening up the oesophagus, 1.5 cm long, sharp impacted bone was found on its anterior wall while the posterior wall displayed a slit-like opening (Fig. 2, Fig. 3). In the posterior mediastinal view, the probing of the oesophageal opening revealed that the impacted bone traversed into the lumen of the aorta with an AEF located just below the level of the arch (Fig. 4).

## 3 Discussion

Complications of oesophageal foreign bodies can be devastating, ranging from mucosal ulceration, oesophageal perforation, primary AEF, mycotic aortic aneurysms, and sepsis due to mediastinitis [7,8]. Oesophagus with its inherent unadaptable properties, peristalsis, and proximity to the thoracic aorta makes it more prone to such complications [9]. Oesophageal foreign bodies represent the second commonest cause (19 %) for AEF [10]. Impacted foreign bodies could lead to pressure necrosis, pseudo aneurysm formation, or inflict direct penetrating injury leading to AEF [11,12].

AEF after foreign body ingestion is very rare. Out of a cohort of 3209 patients presenting with oesophageal foreign bodies to a single specialized center over 4 decades, the incidence was found to be around 1 % [12]. This rarity suggests the need for a high index of clinical suspicion in probable cases aided by the symptoms discussed earlier; however, some patients may have non-specific symptoms or even be asymptomatic [12]. In another study, 59 % of the patients with AEF had a history of mid-thoracic pain and 65 % had a sentinel arterial haemorrhage [10]. A massive haemorrhage strongly points towards AEF. In suspected cases, early investigations are a priority. UGIE is a useful tool that might show the AEF with pulsating mucosal protrusion with a fistula in the center, submucosal protrusion, or ulceration [13]. However, the diagnostic yield may be as low as 25 % [14] and as in our case, uncontrollable bleeding may result in poor visualization. CT studies [15] and CT angiograms were found to have higher rates of diagnosis with rates up to 60 % reported in the latter [14].

The management of a diagnosed or suspected AEF is centered around initial resuscitation (in cases of massive haemorrhage or sepsis), control of bleeding, and definitive therapy. When the bleeding is torrential, the initial placement of the Sengstaken-Blakemore tube had been suggested as means of exerting the tamponade effect to control bleeding [16]. Surgical interventions are essential in AEF. Endovascular procedures (thoracic endovascular aortic repair, TEVAR) are being performed specially in unstable patients. These are less invasive and facilitate faster control of bleeding, with mortality rates around 40 % at 7 months [17]. Since endovascular repair does not deal with the oesophageal component of the AEF, fatal mediastinitis could ensue and recurrent infection and death in the intermediate period are likely [9]. This warrants definite open surgery with aortic repair and concurrent oesophageal reconstruction. On the other hand, TEVAR with video-assisted thoracoscopy surgery has also been successfully utilized by some authors [7]. It should be reiterated that endovascular stenting and open surgery require expertise and equipment, which might not be freely available in low-resource settings as well as advanced imaging modalities before surgery.

Food bolus impaction is not uncommon in adults and 95 % of the time, it is accidental [18]. Toothpicks, animal and fish bones, and steakhouse syndrome are common causes of food bolus impaction in adults. In cases of sharp foreign bodies, an urgent endoscopy study is suggested [19]. While UGIE can aid in locating the foreign body, extraction should be done judiciously as foreign body migration and further injury to the aorta could occur [7]. Informed consent is a necessity before UGIE and the patients with capacity (similar to our patient) who refuse such essential investigations pose a significant obstacle to safe patient management. However rare, patients admitted with foreign body ingestion with a high risk of fatal outcomes need to be preemptively educated on the probable risk. Food preparation practices both at the domestic and industrial levels can also be studied and safe practices can be introduced.

## 4 Conclusion

This case report highlighted a fatal outcome following a primary AEF as a result of a sharp piece of ingested mutton bone. Refusal of UGIE and disclosure of resolution of symptoms by the patient led to the missed opportunity of diagnosis and subsequent formation of AEF and massive gastrointestinal haemorrhage. Further imaging and specialized surgical interventions could have been offered early if urgent UGIE was performed. In cases of high-risk food bolus impaction, institutional protocols are essential in these times of increased consumption of meat-based diets.

## Consent

Written informed consent was obtained from the next-of-kin of the patient for the publication of this case report and accompanying images. A copy of the written consent is available for review by the Editor-in-Chief of this journal on request.

## Provenance and peer review

Not commissioned, externally peer-reviewed.

## Sources of funding

None.

## Ethical approval

Our institution does not require ethical approval for reporting individual cases or case series.

## CRediT authorship contribution statement

Concept, consent, literature review, drafting of the initial and final manuscript, approval of the final manuscript — all authors.

## Research registration (for case reports detailing a new surgical technique or new equipment/technology)

Not applicable.

## Funding

This research did not receive any specific grant from funding agencies in the public, commercial, or not-for-profit sectors.

## Ethical approval

Our institution does not require ethical approval for reporting individual cases or case series.

## Declaration of competing interest

None declared.
